# Water pollution and the brain

**DOI:** 10.1016/j.clinsp.2024.100424

**Published:** 2024-06-28

**Authors:** Fulvio A. Scorza, Feres Chaddad-Neto, Larissa Beltramim, Josef Finsterer, Tomás de la Rosa

**Affiliations:** a Disciplina de Neurociência, Escola Paulista de Medicina/Universidade Federal de São Paulo (EPM/UNIFESP), São Paulo, SP, Brazil; b Ministério do Desenvolvimento Agrário e Agricultura Familiar (MDA), São Paulo, SP, Brazil; c Unidade de Neurocirurgia do Hospital Beneficência Portuguesa, São Paulo, SP, Brazil; d Departamento de Neurologia e Neurocirurgia, Escola Paulista de Medicina/Universidade Federal de São Paulo (EPM/UNIFESP), São Paulo, SP, Brazil; e Neurology and Neurophysiology Center, Vienna, Austria; f Neuroscience Department, Universidad de Cádiz, Facultad de Medicina 3 Planta, Cádiz, Spain; g Instituto de Investigación e Innovación Biomédica de Cádiz (INiBICA), Cádiz, Spain; h Centro de Investigación Biomédica en Red en Salud Mental (CIBERSAM), Instituto de Salud Carlos III, Madrid, Spain

Water is an essential nutrient.^1^, ^2^, ^3^ Water is the main component of cells, tissues, and organs and accounts for 50 %‒70 % of a young adult's body weight.^1^^,^^4^, ^5^, ^6^ For example, a 70 kg human contains 42 liters of total body water, of which 28 liters are intracellular water and 14 liters are extracellular fluids.^1^^,^^4^, ^5^, ^6^, ^7^ In general, water consumption is regulated by thirst.^1^^,^^4^ According to experts, humans consume 20 % to 30 % of water in the form of solid food, while 70 % to 80 % comes from drinks, depending on the food choice and type of drink.^1^^,^^4^^,^^8^ Current guidelines recommend that sedentary adults drink an average of 2 liters of water per day.^1^^,^^2^^,^^4^ Total body water weight varies throughout life and is essential for cellular homeostasis (the ability of cells to keep the organism in constant balance even in the face of external fluctuations).^1^^,^^4^^,^^9^ Consequently, an adequate level of fluid intake is required for the optimal functioning of the systems of the human body such as the central and peripheral nervous system, kidneys, liver, digestive tract, reproductive system, and respiratory and cardiovascular systems.^1^^,^^4^^,^^9^ Without going into detail, the best source of liquids is, surprisingly, the water itself! Although Brazil is privileged because the studied country contains approximately 12 % of all fresh water on the planet, the water sources are contaminated by a wide variety of factors, such as the irregular disposal of industrial waste, garbage, and other urban waste, waste from agriculture, industrial or mining activities and deforestation.^10^ Catastrophically poor water quality has serious impacts on human health, including our brains. The human brain is considered the most complex system in our body.^10^ The brain, which is responsible for our sensations, emotions, thinking, language, and consciousness, consists of 86 billion neurons and weighs about 1.5 kg.^11^^,^^12^ The human brain is in constant change throughout life and is therefore a plastic and adaptable organ.^13^^,^^14^ Unfortunately, the negative consequences of environmental pollutants in drinking water are also associated with a deterioration in brain functions and the possible development of neuropsychiatric diseases.^10^^,^^15^ In fact, a number of industrial processes use large amounts of water, and the wastewater produced by these processes has the potential to pollute residential water supplies.^10^ The chemicals that enter the water supply in this way primarily include inorganic salts and organic compounds, which pose health risks and can have massive neurological effects.^10^ For example, acrylamide in drinking water can cause neurotoxic side effects such as tremors, peripheral neuropathy, and sensory ataxia (difficulty or inability to maintain motor coordination).^10^^,^^16^, ^17^, ^18^ Several epidemiological studies examining dietary intake of acrylamide and cancer risk have shown positive associations with the occurrence of endometrial, ovarian, and renal cell cancers.^10^^,^^16^, ^17^, ^18^ In animal studies, the incidence of brain tumors increased when acrylamide was administered in drinking water, and a similar trend was also evident in studies with humans.^10^^,^^16^, ^17^, ^18^ In addition, cases of poisoning have been observed after drinking water from freshly plastered pipes or wells (plaster made with acrylamide).^10^^,^^16^, ^17^, ^18^ In this way, serious neurotoxic effects have been observed in these individuals, including hallucinations and memory impairment.^10^^,^^16^, ^17^, ^18^ Additionally, it is important to highlight that substances applied to agricultural crops (e.g., pesticides) often drain into water supply sources.^10^ In addition, livestock activities can also introduce animal waste and bacteria into water runoff, and the flow of chemical products from gardens can contribute to water contamination.^10^ In fact, it is well known that pesticide residues have serious effects on human health.^10^^,^^19^^,^^20^ From a neurological perspective, it has been demonstrated that some types of pesticides (e.g., carbamates, organochlorines, and organophosphates) can cause serious irreversible damage to the brain and are considered potential risk factors for the development of neurodegenerative diseases.^10^^,^^19^, ^20^, ^21^ In parallel, several pesticides have harmful neurological effects and indirectly unbalance the cellular mechanisms that maintain brain activity.^19^, ^20^, ^21^ Additionally, exposure of agricultural workers to pesticides increases the incidence of anxiety and depression, which has a major impact on mental health.^19^, ^20^, ^21^ Suicide cases among these individuals rose twice as high as the national average.^19^, ^20^, ^21^ Ironically, another threat to the quality of water reservoirs lies in the practices to improve drinking water quality.^10^^,^^22^^,^^23^ Alumina, a harmful material for bleaching and precipitating organic matter, can increase aluminum levels in water.^10^^,^^22^^,^^23^ Accordingly, epidemiological evidence clearly demonstrates that aluminum levels in drinking water are associated with the occurrence of Alzheimer's disease and that these patients have excessive accumulation of aluminum in the brain tissue.^10^^,^^22^^,^^23^ At the same time, as many people in the world are exposed to other toxic metals in drinking water, there is also great concern in the scientific community about the toxic effects of arsenic, lead, and cadmium on the human brain.^10^^,^^24^^,^^25^ In fact, several studies have shown that chronic consumption of these metals through drinking water leads to changes in cognitive abilities and impaired sensory and motor functions.^10^^,^^24^^,^^25^ Another important aspect is the contamination of water by pharmaceutical waste.^10^^,^^26^ According to health authorities, about half of the population takes at least one prescribed medication every thirty days, and some of these chemicals gradually leak into waterways,^10^^,^^26^ having undesirable consequences on human health. In fact, recent studies have shown that the presence of drug residues in the environment (e.g., food and water for consumption) can have negative effects on brain development and pose a serious threat to the health and lives of current and future generations.^10^^,^^26^ Although each of the environmental pollutants present in water has a distinctive neurotoxicity profile, inflammatory and oxidative mechanisms play a role important in the brain changes caused by most of these chemicals.^10^^,^^27^^,^^28^ In this sense, the introduction of therapeutic strategies that reduce absorption, increase excretion and prevent or minimize the harmful effects of environmental pollutants present in water or tissues and organs is fundamental today.^10^^,^^29^^,^^30^ In general, water pollution must be viewed as a serious public health problem and ensuring the supply of safe drinking water for all is a challenging task.^31^ In that regard, the appropriate and effective implementation of public policies to deal with water pollution problems is important. In addition, the development of educational campaigns is extremely necessary to provide our population with accurate and up-to-date information that water is a scarce resource and that consuming or ingesting contaminated water causes serious harm to our mental and organic health.

## Authors’ contributions

All authors contributed equally to the conceptualization, writing, and reviewing of the present manuscript.

## Funding

Our studies are supported by the following grants: FAPESP (Fundação de Amparo à Pesquisa do Estado de São Paulo); CNPq (Conselho Nacional de Desenvolvimento Científico e Tecnológico), Coordenação de Aperfeiçoamento de Pessoal de Nível Superior (CAPES) and ‘Juan de la Cierva’ grant financed by (MICIU/AEI/10.13039/501100011033) and by European Social Fund (FSE+)

## Conflicts of interest

The authors declare that the research was conducted in the absence of any commercial or financial relationships that could be construed as a potential conflict of interest.
